# College and Hospital Notes

**Published:** 1903-11

**Authors:** 


### COLLEGE AND HOSPITAL NOTES.

The Massachusetts General Hospital formally opened its new outpatient department on "Ether Day," October 16, 1903. Invitations were issued by the trustees to the friends of the hospital, to inspect the new branch and other recent additions on the day mentioned between 12 and 3 o'clock. Refreshments were served on the lawn at 1 o'clock, to a large gathering of people, many of whom came from other places.

---

The medical department of the University of Buffalo opened its fifty-eighth regular session Monday evening, September 28, 1903. Dr. Matthew D. Mann, dean of the faculty, presided, and Dr. Carlton C. Frederick gave the inaugural lecture, the trend of which was to indicate how success might be attained in the profession of medicine. In addition to the faculty there were a number of physicians from out of town in attendance. The dean said that the department was in better shape as to equipment than ever before and that its future was very bright.

